# Supplementary material for: Minimal Hepatic Encephalopathy Affects Daily Life of Cirrhotic Patients: A Viewpoint on Clinical Consequences and Therapeutic Opportunities
Source: J Clin Med. 2022 Dec 6;11(23):7246. doi: 10.3390/jcm11237246 (PMC9736966; doi:10.3390/jcm11237246)
Supplement: Supplementary file 1 [file jcm-11-07246-s001.zip › jcm-2024530-supplementary.pdf]

Supplementary Table S1: diagnostic tests for MHE [2].

**Test**	**Tested domain**	**Copyright**	**Dedicated (Europe-Asia/USA)**	**Time required for administration and interpretation (min)**	**Comments**
NCT-A	Psychomotor speed	Yes	No/No	1-2	Poor specificity
NCT-B	Psychomotor speed, set shifting, divided attention	Yes	No/No	1-3	Poor specificity
BDT	Visuospatial reasoning, praxis, psychomotor speed	Yes	No/Yes	10-20	It can be used for dementia testing as well
DST	Psychomotor speed, attention	Yes	No/Yes	4	Tends to be very sensitive and is an early indicator
LTT	Psychomotor speed, visuomotor ability	Yes	No/Yes	2-4	Outcomes are errors and time; tests a balance between speed and accuracy
SDT	Psychomotor speed	Yes	No/es	1-2	Only tests psychomotor speed but has a higher sensitivity than DST
PHES	Psychomotor speed, set shifting, attention, visual perception and visuospatial orientation, visuomotor ability	Yes	No/Not for all tests	15	Easy to administer, good external validity; performance influenced by age and education
R-BANS	Verbal/visual/working memory, visuospatial, language, and psychomotor speed	Yes	No/Yes	35	Has been primarily studied in dementia and brain injury. Limited HE experience

Animal-naming test	Semantic fluency test, verbal retrieval and recall	No	No	1	Easy tool for caregivers for identify mental status alterations, for illiterate patients,
Inhibitory Control Test (ICT)	Response inhibition, working memory, vigilance, attention	Yes	No/No	15-20	Need highly functional patients, familiarity with computers may be needed
SCAN test	Working memory, vigilance, attention	Yes	No/No	15-20	Prognostic value (predictive of survival)
Continuous Reaction Time (CRT)	Motor reaction speed, sustained attention, inhibitory control	NA	NA	10	Not affected by age, educational level and intelligence; no learning effect; simple software and hardware are required
Stroop Test	Psychomotor speed, cognitive flexibility, functioning of anterior attention system	No		5	Highly accessible by web (available in app-form); quick to teach to subjects and simple to score and interpret; sensitive to cognitive change in MHE
Critical Flicker Frequency	Measure of visual temporal resolution	NA	NA	May vary	Predictor of mortality; associated with the development of overt HE; requires specialized equipment
EEG	Generalized brain activity	No	Yes/Yes	10-15	Can be performed in comatose patients
Visual evoked potentials	Interval between visual stimulus and activity	No	Yes/Yes	May vary	Highly variable and poor overall results
Brainstem auditory evoked potentials	Response in the cortex after auditory click stimuli	No	Yes/Yes	May vary	Inconsistent response with HE testing/prognostication

P300 cognitive evoked potentials	An infrequent stimulus embedded in irrelevant stimuli is studied	No	Yes/Yes	Different ranges	Correlates with the severity of the hepatic encephalopathy (prolongation of P300 latencies and reduction in amplitude)
CFF	Visual discrimination and general arousal	No	Yes/Yes	10	Need highly functional patients

Supplementary Table S2: published studies on treatment of minimal hepatic encephalopathy.

First Author/ Journal	Year	Study type	MHE diagnosis	Active treatment(s)	Patients treated	Length of treatment	Objectives	Main results
**Psycometry/ MHE reversal**								
Watanabe et al. [70]	1997	Original, randomized	NCT A, DST, BDT	Lactulose	36 (22)	8 w	Psychometry	MHE disappeared in 10 (50%) of the 20 treated patients and persisted in 11 (85%) of the untreated 13 patients.
Horsmans et al. [88]	1997	Original, randomized	NCT, RTT, sinusoid test, psychomotor performance tests	Lactulose	14 (7)	2 w	Psychometry, ammonia level	NCT (71% vs 14%) and RTT (86% vs 60%) significantly improved; ammonia levels significantly decreased (71% vs 14%).
Dhiman et al. [71]	2000	Original, randomized	NCT A, NCT B, FCT A, FCT B, PC, BDT	Lactulose	18 (10)	12 w	Psychometry	Psychometric tests significantly improved (80% vs 0%)

Malaguarnera et al. [89]	2007	Randomized control study	TMT, WAIS-R, BDT, SDMT, MMSE, EEG	Bifidobacterium longum plus fructo-oligosaccharides (FOS) vs placebo	60 (30)	90 d	Psichometry, ammonia level	Ammonia level, TMT-A and TMT-B times significantly decreased (p=0.000); performance on SDMT (p < 0.05), MMSE (p=0.000) and BDT (p=0.000) significantly improved.
Sharma et al. [82]	2008	Original, randomized	NCT A, NCT B, FCT A, FCT B	Lactulose or Probiotic or Lactulose +Probiotic	105 (35/35/35)	4 w	Psychometry, P300 ERP, ammonia level	Psycometric tests significantly improved in more than 50% of patients:<br>- Lactulose group: 19/31 (61%)<br>- Probiotic group: 15/29 (52%)<br>- Combination group: 20/30 (67%)
Malaguarnera et al. [90]	2008	Randomized controlled study	TMT, SDMT, AVL, MMSE	Acetyl-L-carnitine	125 (65)	90 d	Psychometry	Psycometric tests (TMT A, TMT B, MMSE, SDMT and AVL) significantly improved (p<0.01).
Sharma et al. [91]	2014	Randomized control trial	NCT-A, FGT-A, DST, CFF	LOLA or Rifaximin or probiotics or placebo	124 (31/31/32)	8 w	Psychometry and CFF	Psychometric tests and CFF significantly improved after treatment (67.7%, 70.9%, 50% vs 30%, p<0.05).
Zhang, Y et al [74]	2015	Original	NCT A, NCT B, DST	Rifaximin	26	1 w	Psychometry, ammonia level, SIBO	Psychometry significantly improved in 58% (15/26). SIBO ameliorated in 76% (13/17) and ammonia level decreased, mostly in those with SIBO.
Xia et al. [92]	2018	Randomized control trial	NCT-A, DST	Probiotics	67 (30)	12 w	Psychometriy, intestinal microbiota, ammonia level, intestinal mucosal barrier function.	Psychometric tests significantly improved (NCT-A, DST).<br>Ammonia level significantly decreased (76.4 $\pm$ 37.3 vs. 152.0 $\pm$ 48.3 umol/mL, p=0.032).<br>The parameter of intestinal barrier (LPS, D-lactate).
**Quality of life**								

Prasad et al. [8]	2007	Original, randomized	NCT A, NCT B, FCT A, FCT B, PC, BDT	Lactulose	70 (45)	12 w	Psychometry, HRQOL	The mean total SIP score significantly improved among patients in the treated group (10.39 vs 3.77) compared with patients in the untreated group. Improvement in HRQOL was related to the improvement in psychometry.
Sidhu et al. [80]	2011	Original, randomized	NCT A, FCT A, DST, BDT, PC	Rifaximin	94 (49)	8 w	MHE reversal, HRQOL	MHE reversal in 75.5% (37/49) vs 20% (9/45). The mean total SIP score improved significantly in rifaximin group and this correlated with improvement in psychometry.
Mittal et al. [81]	2011	Original, randomized	NCT A, NCT B, FCT A, FCT B, PC, BDT	Lactulose or Probiotics or LOLA	160(40/40/40)	12 w	Psychometry, ammonia level, HRQOL	MHE significantly improved in all three treatment groups compared with no treatment (P = 0.006).<br>The SIP score significantly decreased in treatment groups vs placebo (p<0.001) and this correlated with improvement in psychometry on multivariate analysis.
Bajaj et al [93]	2014	Original, randomized	NCT A NCT B, DST, BDT	Probiotics	30 (14)	8 w	Psychometry, ammonia level, inflammatory markers, HRQOL	No effects on psychometry but reduction in inflammatory markers (endotoxin and TNF-alpha).
Sidhu et al. [76]	2016	Original, randomized	NCT A, FCT A, DST, BDT, PC	Lactulose vs. Rifaximin	112 (55/57)	12 w	MHE reversal, HRQOL	Recovery from MHE in 69 % (38/55) and 74% (42/55).<br>HRQOL significantly improved in both groups, but with more side effects in the lactulose group.
Luo et al. [73]		Meta-analysis	Different diagnostic tools	Lactulose	434	Variable	Psychometry, ammonia levels, HRQOL, progression to OHE	Lactulose reduced the mean number of abnormal neuropsychological tests (RR=0.52), ammonium levels and risk of developing OHE (RR=0.17). HRQOL significantly improved.

Malaguarnera et al. [94]	2018	Randomized control trial	NCT-A, NCT-B, SF-36, BDI, STAI	Resveratrol	90 (35)	90 d	HRQOL, depression, anxiety	BDI ($p < 0.001$) and STAI ($p < 0.001$) significantly decreased in the treatment group.<br>Physical function, body pain, general health and social function significantly improved after therapy.
Wang et al. [95]	2019	Randomized control trial	NCT-A, DST	Lactulose	98 (67)	60 d	Psychometry, HRQOL, intestinal microbiota	MHE reversal was significantly higher in the treatment group (RR=0.46, p= .0002) and physical function significantly improved (p= 0.0212).
Fagan et al [87]	2022	Randomized control trial	Stroop test, PHES, CFF	Albumin	48 (24)	5 w	Psychometry, HRQOL	Albumin treatment determined MHE reversal and amelioration of HRQOL (physical functioning)
**Driving ability**								
Bajaj et al. [83]	2011	Original, randomized	NCT A, NCT B, DST, BDT, ICT	Rifaximin	42 (21)	8 w	Psychometry, HRQOL, driving ability, anti-inflammatory interleukins	Psychometry, driving errors, speeding and illegal turns significantly improved after treatment.<br>The SIP score improved in the psychosocial dimension.
**OHE development**								
Bajaj et al. [86]	2008	Original, randomized	NCT A, DST, BDT	Probiotic yogurt	25 (17)	8 w	MHE reversal, OHE development, HRQOL, Ammonia level, cytokines	MHE recovery in 71% vs 0% (p=0.003). OHE developed in 0% vs 25% (p=0.1)<br>No difference in HRQOL, ammonia level and cytokines.
**Risk of falls**								
Roman et al. [85]	2019	Original, randomized	PHES	Probiotic	36 (18)	12 w	Psychometry, TUG, gait speed, cytokines	Psychometry, gait speed and TUG significantly improved after therapy. Pro-inflammatory cytokines significantly

								decreased (C-reactive protein, tumor necrosis factor alpha, claudin-3).
**Sleep disorders**								
Singh et al. [42]	2017	Prospective	PHES, CFF	Lactulose	50 (50)	12 w	Psychometry, ammonia level, PSG, PSQI, HRQOL	Psychometry and sleep parameters improved after therapy.

MHE, Minimal Hepatic Encephalopathy; **OHE**, Overt Hepatic Encephalopathy; **HRQOL**, Health Related Quality of Life; **NCT-A**, number connection test-A; **NCT-B** number connection test-B; **BDT**, block design test; **SDT**, serial dotting test; **DST**, digit symbol test; **LTT**, line tracing test; **PHES**, psychometric hepatic encephalopathy score; **ICT**, inhibitory control test; **CFF**, critical flicker frequency; **EEG**, electroencephalogram; **ICT,** Inhibitory Control Test; **BCAA**, Branched Chain Amino Acids; **SIBO**, Small Intestine Bacterial Overgrowt; **CEP**, Cognitive Evoked Potential; **AVL,** verbal learning test; **FCT,** figure connective tes; **RTT,** race track test; **PC,** picture completion; **WAIS-R,** Wechsler Adult Intelligence Scale—Revised; **SDMT** symbol digit modalities test; **BDI**, Back depression Inventory, **STAI**, stay trait anxiety inventory. **PSG**, polysomnography. PSQI, Pittsburgh Sleep Quality Index.
